# The Dental Profession

**Published:** 1886-05

**Authors:** 


### "THE DENTAL PROFESSION."

Many dentists, who conscientiously believe that dentistry is a specialty in medical science, are accustomed to speak of "the dental profession," "our profession," "the profession of dentistry," etc., in seeming ignorance of the fact that they thus separate it entirely from medicine. We frequently hear the claim advanced that dentistry is as much a part of general medical science as is Ophthalmology, or Gynecology. But who ever heard a specialist of either of these branches speak of "the profession of ophthalmology" or the "profession of laryngology?" Practitioners of these departments belong to the profession of medicine because they hold the medical degree, and not because they are specialists. Photographers and actors speak of their "profession," and thus admit that they follow separate and distinct callings. It is improper to speak of Dermatology as a "profession." It belongs to the profession of Medicine, but its practitioners are not admitted or acknowledged unless they have first graduated in medicine and hold the medical degree.

If dentistry is to be acknowledged as a department of medicine, all acknowledged dentists must have a medical diploma. No man will be accepted as a medical man without it, no matter what his attainments or knowledge may be. Medicine knows no diploma save that of a medical college. Ophthalmologists and Gynecologists have no distinct degree. If they had, they would separate themselves from the mother profession and form a profession of their own. Dentists who desire to be acknowledged as practicing a department in medicine must cease to speak of "the profession of dentistry," and enter the medical ranks in a regular manner. Those who believe that their vocation is an independent one, which should stand on its own merits, do right in alluding to "the profession of dentistry," and in sustaining our own separate schools and degree. Let us be consistent, and when we speak of "the profession of dentistry" comprehend what we are saying.

